# Effect of Having Concurrent Mutations on the Degree of Aggressiveness in Patients with Thyroid Cancer Positive for *TERT* Promoter Mutations

**DOI:** 10.3390/cancers15020413

**Published:** 2023-01-08

**Authors:** Sama Alohali, Alexandra E. Payne, Marc Pusztaszeri, Mohannad Rajab, Véronique-Isabelle Forest, Michael P. Hier, Michael Tamilia, Richard J. Payne

**Affiliations:** 1 Department of Otolaryngology—Head and Neck Surgery, King Faisal Specialist Hospital & Research Center, Riyadh 11564, Saudi Arabia; 2 Departments of Otolaryngology—Head and Neck Surgery, Royal Victoria Hospital, McGill University, 3755 Côte-Sainte-Catherine Road, Montreal, QC H3T 1E2, Canada; 3 Departments of Otolaryngology—Head and Neck Surgery, Jewish General Hospital, McGill University, 3755 Côte-Sainte-Catherine Road, Montreal, QC H3T 1E2, Canada; 4 Health Sciences, Marianopolis College, Montreal, QC H3T 1E2, Canada; 5 Department of Pathology, Jewish General Hospital, McGill University, Montreal, QC H3T 1E2, Canada; 6 Department of Otolaryngology—Head and Neck Surgery, King Faisal Specialist Hospital & Research Center, AlMadinah AlMunawwarah 42523, Saudi Arabia; 7 Department of Medicine—Endocrinology, McGill University, Montreal, QC H3T 1E2, Canada

**Keywords:** molecular testing, thyroid cancer, *TERT* promoter mutations, *BRAF*, *RAS*

## Abstract

**Simple Summary:**

As molecular testing of thyroid nodules becomes more common, thyroid specialists must be able to interpret and understand the clinical implications of the results. A telomerase reverse transcriptase (*TERT*) promoter mutation can strongly predict thyroid cancer aggressiveness. However, the reason why some thyroid cancers with *TERT* promoter mutations are more aggressive than others remains unclear. This study aimed to examine whether *TERT* promoter mutations coexisting with other mutations are linked to more aggressive disease than *TERT* promoter mutations alone. The medical records of patients who had thyroid surgery and *TERT* promotor mutations were examined. Our findings showed that the likelihood of aggressive thyroid cancers was 10 times higher in patients with *TERT* promoter and other concurrent mutations. Thyroid specialists can use our results to accurately interpret the molecular testing of thyroid nodules, provide appropriate counseling, and discuss possible management options accordingly.

**Abstract:**

This study aimed to examine whether concurrent mutations with a *TERT* promoter mutation are associated with a greater likelihood of more aggressive disease than a *TERT* promoter mutation alone. The medical records of 1477 patients who underwent thyroid surgery at two tertiary hospitals between 2017 and 2022 were reviewed. Twenty-four patients had *TERT* promoter mutations based on molecular profile testing. Clinicodemographic data, mutational profiles, and histopathological features were assessed. Descriptive analysis, Fisher’s exact test, and binary logistic regression were performed. Seven patients had single-gene *TERT* promoter mutations, and 17 had concurrent mutations, including *BRAF V600E*, *HRAS*, *NRAS*, *PIK3CA*, and *EIF1AX*. The overall prevalence of malignancy was 95.8%, of which 78.3% were aggressive thyroid cancers. There was a statistically significant association between concurrent mutations and disease aggressiveness. The odds of having aggressive disease were 10 times higher in patients with a *TERT* promoter mutation and a concurrent molecular alteration than in those with a *TERT* promoter mutation alone. This is an important finding for thyroid specialists to consider when counseling patients concerning risk stratification and management options.

## 1. Introduction

The incidence of thyroid cancer has increased rapidly in the last few decades, and it is expected to be the fourth most common cancer by 2030. This significant rise is mainly attributed to advances in imaging modalities that can easily detect thyroid nodules and, therefore, thyroid cancer. Although thyroid cancer has an excellent prognosis in most cases, it is estimated that up to 20% of thyroid cancer cases will recur, up to 30% will metastasize regionally, less than 4% will metastasize to distant organs, and a small percentage will result in mortality (0.4%) [1,2,3,4]. Therefore, it is important to establish prognostic indicators of disease aggressiveness to guide risk stratification and to determine the extent of surgery and adjuvant treatment regimens for thyroid cancer. To date, no single factor has been attributed to thyroid cancer outcomes; instead, a group of factors are known to affect prognosis. Several factors, including patient characteristics, mutational profiles, and histopathological features, have been identified and described in the literature.

As molecular testing of thyroid nodules becomes more common, it is of paramount importance for thyroid specialists to be able to interpret and understand the clinical implications of the results. Several studies have established the diagnostic values and risk stratification abilities of different molecular profile tests for thyroid cytology specimens. To decrease the substantial number of unnecessary thyroid surgeries performed worldwide, molecular profile tests aim to differentiate patients who are more likely to require surgery from those more likely to require conservative management. Validation studies using the ThyroSeq v3 testing platform have confirmed its ability to rule in disease with a positive predictive value of 66% and rule out disease with a negative predictive value of 97% compared with ThyGeNEXT, which has a positive predictive value of 97% and a negative predictive value of 75% [5,6]. It should be emphasized that these percentages are not fixed and are essentially dependent on each institution’s disease prevalence.

Abnormal activation of the mitogen-activated protein kinase (MAPK) signaling pathway has been described in carcinogenesis in multiple body sites, including the thyroid. Many fundamental mutations in thyroid cancer target different constituents of the MAPK signaling pathway, including *BRAF V600E* and *RAS* gene point mutations, *RET::PTC* and *PAX8::PPARγ* chromosomal rearrangements, and telomerase reverse transcriptase (*TERT*) promoter mutations. Varying levels of growth factors, hormones, and cytokines interact with cell surface receptor tyrosine kinases in the MAPK signaling pathway, which is responsible for regulating cell proliferation, differentiation, and apoptosis [7,8].

*TERT* is a protein subunit of telomerase that adds telomeres to the ends of chromosomes to maintain their length. It is mainly found in germ lines and stem cells and only rarely in most human somatic cells. The addition of telomeric repeats to the ends of chromosomes in each cell cycle prevents cell death. Enhancing the telomerase function in cancer allows cancer cells to obtain “replicative immortality”. *TERT* promoter mutations exist in human cancer cells responsible for melanoma, bladder cancer, and glioblastoma [9,10,11,12]. X Lie et al. reported, for the first time, the detection of *TERT* promoter mutations in thyroid cancer. Moreover, the mutation was not present in benign thyroid tumors (0 out of 85 patients) [13]. The prevalence of *TERT* promoter mutations in papillary thyroid cancer is estimated to be between 5% and 25%, varying among subtypes. It is more common in aggressive subtypes, such as the tall cell variant [14]. The prevalence of *TERT* promoter mutations in benign thyroid tumors, follicular thyroid cancer, poorly differentiated thyroid cancer, and anaplastic thyroid cancer is estimated to be 0%, 20%, 20–50%, and 30–75%, respectively. In one study, the prevalence was significantly higher in tumors with aggressive histological features (32.7%) than in those with nonaggressive histological features (15.3%) [15].

The modified initial risk stratification system for differentiated thyroid cancer published in the American Thyroid Association (ATA) 2015 guidelines recommends using the *TERT* promoter mutation status, if available, to improve risk estimates [16]. Many studies have revealed that a *TERT* promoter mutation is a predictor of thyroid cancer aggressiveness [8,17]. In their systematic review and meta-analysis, Yin et al. reported significant associations with *TERT* promoter mutations: lymph node metastasis, extrathyroidal extension, distant metastasis, poor outcome (persistent or recurrent disease), and mortality [18]. Additionally, Ebina et al. identified 133 patients with *TERT* promoter mutations who exhibited significantly worse 10-year cause-specific survival (73.7% vs. 98.1%) and 10-year disease-free survival (53.7% vs. 93.3%) compared to those without mutations. However, only seven patients exhibited a *TERT* promoter mutation alone [19].

Why some thyroid cancers with *TERT* promoter mutations tend to be more aggressive remains unclear. It has been shown that anaplastic thyroid carcinoma possesses a higher number of genetic alterations than papillary thyroid carcinoma, which can be rationalized by the widely accepted progression theory of cancer that implies an ongoing buildup of mutations during progression from differentiated to dedifferentiated cancer [20]. Recent meta-analyses have revealed that thyroid cancers with concurrent *BRAF V600E* or *RAS* and *TERT* promoter mutations were associated with increased tumor aggressiveness compared to those harboring *BRAF V600E*, *RAS*, or *TERT* promoter mutations alone [21]. However, most previous studies did not account for the presence of other rare mutations that may also influence the disease outcomes of thyroid cancer with *TERT* promoter mutations. The present study aimed to examine whether concurrent mutations with *TERT* promoter mutations are associated with more aggressive thyroid cancers compared to *TERT* promoter mutations alone. We hypothesized that the presence of any other molecular alterations, hence the accumulation of mutations during carcinogenesis, would influence the aggressiveness of thyroid cancer with *TERT* promoter mutations. To our knowledge, this is the first study to evaluate the role of coexisting mutations, taking into account all possible mutations available in the commercial molecular tests (ThyGeNEXT or ThyroSeq v3) in patients with thyroid cancer positive for *TERT* promoter mutations in the development of aggressive features.

## 2. Materials and Methods

### 2.1. Study Participants and Data Collection

This multi-center study was approved by the McGill University Health Centre (MUHC) Research Ethics Committee (File Number: MP-37-2021-7665). A multi-center retrospective chart review of 1477 patients who underwent thyroid surgery at two university teaching hospitals in Montreal, Canada, between January 2017 and May 2022 was performed. The study included adult patients who underwent ultrasound-guided fine needle aspirations of dominant thyroid nodules and tested positive for one of the two *TERT* promoter mutations located at hotspots chr5, 1,295,228 C  >  T (C228T) and 1,295,250 C  >  T (C250T) in molecular profile testing. Clinical and demographic data, such as age, sex, Bethesda category of thyroid nodules, genetic alterations on molecular profile testing, and presence of other molecular mutations, were collected. The types of treatment and extent of thyroid surgeries were also noted. Thyroid nodules were categorized as benign or malignant based on postoperative histopathological findings. The Strengthening the Reporting of Observational Studies in Epidemiology checklist was used to guide the reporting of this study.

### 2.2. Molecular Profile Testing Technique

Informed consent was obtained from all patients before molecular profiling. Fine needle aspiration was performed in the clinic under ultrasound guidance by fellowship-trained Otolaryngology—Head and Neck surgeons. An alcohol swab was used for skin preparation, followed by injection of the subcutaneous tissue with xylocaine 1% epinephrine 1/100,000 concentration. A 21-gauge needle with a beveled tip measuring 1.5 inches and a 10 cc syringe with a luer lock tip were used. Ultrasound guidance using a linear high-frequency transducer in a parallel technique was used to identify the most suspicious nodule and obtain the biopsy. To improve the quality of the biopsy, the suction effect was usually maintained throughout sampling by pulling back the plunger by 1 to 2 cc. Samples were then analyzed at a commercial laboratory (Interpace Diagnostics) at the University of Pittsburgh Medical Center, Pittsburgh, PA, USA, for ThyroSeq v3^®^ testing, or Parsippany, NJ, USA, for ThyGeNEXT^®^ testing.

### 2.3. Aggressive Thyroid Cancer

The thyroid cancer was classified as aggressive if it exhibited at least one of the following features: evidence of aggressive papillary carcinoma variants (tall cell, hobnail, solid, diffuse sclerosing), poorly differentiated carcinoma, extensive vascular invasion, gross extrathyroidal extension, and presence of regional lymph node or distant metastases. The predefined criteria were based on methods used in recent publications by our group of researchers [22]. Features on histopathology used to define aggressive disease would generally warrant a more aggressive standard treatment approach according to the 2015 ATA guidelines [16], including total thyroidectomy, neck dissection, and adjuvant radioactive iodine, as opposed to hemithyroidectomy alone. Anaplastic carcinoma was excluded due to its rarity and the design of the study.

### 2.4. Statistical Analysis

Descriptive statistics are reported as means ± standard deviations (SDs). An independent-sample *t*-test was used to compare the mean age and tumor size between patients with single-gene *TERT* promoter mutations versus those with concurrent mutations. Fisher’s exact test was used for categorical variables to assess the associations among thyroid cancer aggressiveness, sex, Bethesda scores, and other mutations. Binary logistic regression was performed to quantify the association between the presence of co-mutations and thyroid cancer aggressiveness. Two-sided *p*-values < 0.05 were considered to indicate statistical significance.

## 3. Results

### 3.1. TERT Promoter Mutation Detection Rate

This was a chart review of 1477 patients who underwent thyroid surgery. Of those, 720 had undergone molecular testing, and 24 tested positive for the *TERT* promoter mutation. Therefore, the detection rate in our sample was 24/720 (3.33%).

### 3.2. Patients’ Characteristics and Molecular Profile Distribution

Table 1 summarizes the baseline patient characteristics. Most patients were female (75%), and the mean age of the patients was 68 years. The minimum and maximum ages at the time of surgery were 53 and 83 years, respectively.

**Figure 1:**
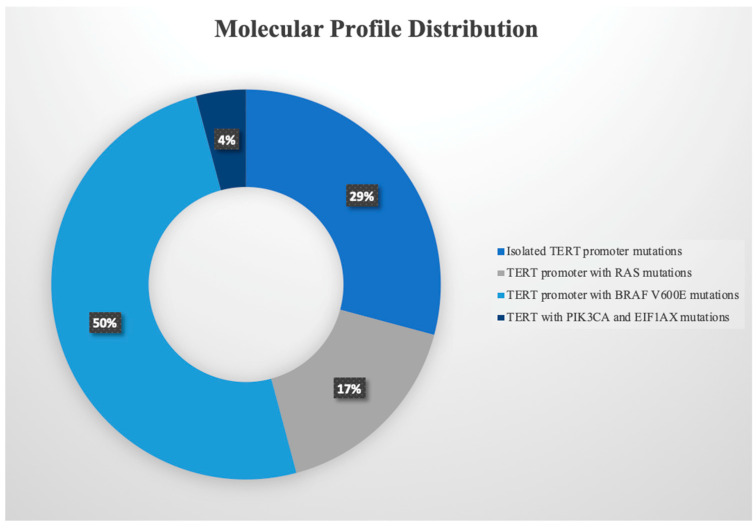
Mutation profile distribution of patients who underwent ultrasound-guided fine needle aspiration of a dominant thyroid nodule and tested positive for a *TERT* promoter mutation using ThyGeNEXT^®^ or Thyroseq V3^®^.

The mean size of the dominant thyroid nodule was 3.7 cm. The minimum and maximum nodule sizes on the final pathological examination were 1 cm and 7.8 cm, respectively. The frequencies of each cytological category are summarized in Table 1. Figure 1 shows the molecular profile distribution. Single-gene *TERT* promoter mutations were found in seven patients on molecular testing. The remaining 17 patients had concurrent mutations, including *BRAF V600E* (in 12 patients), *RAS* (in four patients: three *NRAS* and one *HRAS*), or *PIK3CA* and *EIF1AX* (in one patient) mutations. Eighty-three percent of patients underwent total thyroidectomy. The overall prevalence of malignancy was 95.8%. All patients, except one, had malignant disease on postoperative histopathological examination. The sole patient with benign disease had a Bethesda category of IV and a single-gene *TERT* promoter mutation on molecular profile testing. Among the remaining 23 patients with malignancy, 18 had at least one of the predefined aggressive features.

### 3.3. Effect of Mutational Profile on Age, Sex, Thyroid Nodule Size, and Bethesda Category

Patients with single-gene *TERT* promoter mutations were significantly older by 13.6 years than those with co-mutations, with mean ages of 77.6 (SD: 4.7) and 64 (SD: 9) years, respectively (*p*-value of 0.001). There was no statistically significant association between mutational status and sex. Furthermore, no statistically significant difference was found between the mean dominant thyroid nodule size of patients with single-gene *TERT* promoter mutations versus those with concurrent mutations (4.16 cm ± 1.77 versus 3.64 cm ± 1.61, *p*-value > 0.05). Finally, the mutational status association with the Bethesda category reached statistical significance; the presence of concurrent mutations was associated with a higher Bethesda category of 5 or 6, as opposed to a Bethesda category of 3 or 4 (*p*-value of 0.009).

### 3.4. Effect of Mutational Profile on Degree of Aggressiveness

The results of Fisher’s exact test are presented in Table 2. The Bethesda categories and molecular profiles were significantly associated with disease aggressiveness, with *p*-values of 0.001 and 0.038, respectively. Of the patients who had aggressive disease on postoperative histopathological examination, 83.3% had co-existing mutations, as opposed to 16.7% of those with a *TERT* promoter mutation alone. On the other hand, of those who had nonaggressive disease on postoperative histopathological examination, 33.3% of patients had co-existing mutations, as opposed to 66.7% of those with a *TERT* promoter mutation alone. Binary logistic regression was performed to quantify the association between concurrent mutations and aggressiveness, revealing statistical significance with a *p*-value of 0.0318 and an odds ratio of 10 (1.22, 81.81) (coefficient 2.3, standard error 1). Fisher’s exact test was also used to assess the relationship between the mutational profile and lymph node status. The presence of concurrent mutations was significantly associated with lymph node status, with a *p*-value of 0.0059. All seven patients with single-gene *TERT* promoter mutations had no positive lymph nodes on final histopathology. Due to the small sample size, subgroup analysis for each pathology type and distant metastasis was impossible. In our study sample, sex was not significantly associated with disease aggressiveness. Nonetheless, all male patients had aggressive disease.

## 4. Discussion

This study supports the hypothesis that thyroid nodules positive for both *TERT* promoter mutations and other mutations are more likely to harbor aggressive features than thyroid nodules positive for *TERT* promoter mutations alone. Furthermore, the odds of having aggressive disease were 10 times higher in patients with *TERT* promoter mutations in addition to concurrent mutations than in those with *TERT* promoter mutations alone.

In our study, more than half of the patients with single-gene *TERT* promoter mutations had nonaggressive features. Moreover, one patient with a Bethesda category IV thyroid nodule had benign disease on final pathology. This observation led us to attempt to recognize potential factors that may influence the degree of aggressiveness of thyroid cancer with *TERT* promoter mutations to more accurately identify patients with poor prognoses.

Of the studies examining *TERT* promoter mutations in patients with follicular adenomas, the reported occurrence is ~0% [13,23,24]. In fact, out of the 552 cases of follicular adenomas studied in these different series, only one study reported a positive case for a *TERT* promoter mutation. Wang et al. found one patient expressing a C228T mutation with a postoperative histopathological diagnosis of follicular adenoma. Of note, the single patient described in the literature subsequently died from metastatic follicular thyroid carcinoma [25]. This supports the theory that a *TERT* promoter mutation may be the initial genetic event in carcinogenesis that promotes tumor cells to acquire other aggressive mutations. Thyroid carcinogenesis occurs through a cascade of steps that requires multiple genetic alterations. The cancer process is initiated by driver mutations, followed by a cascade of secondary mutations, leading to the progression from differentiated to undifferentiated cancer cells. However, the precise timing of when the *TERT* promoter mutation occurs is uncertain. Hence, whether it is a driver mutation or a secondary mutation has yet to be determined [26,27].

When a *TERT* promoter mutation is identified in a malignancy without another identified mutation, it is probable that the *TERT* promoter mutation is promoting the progression of a mutation that is not yet detectable with the currently available molecular tests. As a result, when a *TERT* promoter mutation is the only mutation detected, it is likely that other mutations are present but not detected. Numerous commercial molecular tests are available. These include ThyroSeq v3, Afirma GSC/XA, and ThyGenNEXT/ThyraMIR. The two tests used in our study were ThyroSeq v3 and ThyGenNEXT/ThyraMIR. One hundred and twelve thyroid-related genes are evaluated routinely by the next-generation sequencing assay in the third version of the ThyroSeq test. ThyGenNEXT incorporates a mutation panel consisting of the most common thyroid cancer-related mutations, along with the ThyraMIR microRNA classifier test. Although these tests can widely screen for molecular alterations, ranging from gene fusions, copy number alterations, and gene expression alterations, more accurate molecular profile testing methods are required to better predict disease outcomes [28,29].

Many studies have consistently demonstrated a significant association between *TERT* promoter mutations and older patients. In a recent meta-analysis, the mean patient age was 59.2 ± 15.5 years versus 44.9 ± 15.6 years in patients with *TERT* promoter mutations versus those without such mutations [8]. In our study population, the mean age at the time of surgery was 68 ± 10 years. Additionally, the seven patients with *TERT* promoter mutations alone were significantly older than the 14 patients who had concurrent mutations (77.6 ± 4.7 years versus 64 ± 9 years, respectively). Miyauchi et al. showed that the age at diagnosis could be used to assess lifetime disease progression probabilities, which were 8% for patients in their 60s and 4% for patients in their 70s. This is another reason to support the hypothesis that patients with thyroid cancer positive for *TERT* promoter mutations may not have aggressive disease. [30,31]

We detected an association between poorer prognoses and *TERT* promoter mutations when they coexist with other mutations, highlighting the supportive role of concurrent mutations in developing aggressive thyroid cancer. It has been ascertained that *RAS* oncogene mutations exhibit a low-risk phenotype, with a reported malignancy prevalence of 47%. This prevalence reaches 100% when nodules are positive for a *BRAF V600E* mutation [32]. Furthermore, Radkay et al. demonstrated that the risk of cancer is highly dependent on the *RAS* mutation subtype, with *KRAS* (41.7%) being associated with the lowest risk, followed by *NRAS* (86.8%) and *HRAS* mutations (95.5%) [33]. Four patients in our sample exhibited either *NRAS* or *KRAS* mutations. All had confirmed malignancy, with a 75% incidence of aggressive disease. Zhao et al. studied the significance of concurrent mutations in thyroid cancer. Their findings showed that *TERT* + *RAS* was highly associated with distant metastases and mortality. This is consistent with our findings [8]. Two of our study patients had distant metastases from their thyroid cancer. Both patients had a mutation in one of the *RAS* proto-oncogenes; one metastasized to the mandible and the other to the chest wall, mediastinum, and heart. This observation again validated the synergistic effects of the different genetic mutations, demonstrating their greater impact on disease prognosis.

Our study was also in keeping with other studies in the literature that showed poor clinicopathological outcomes for papillary thyroid cancer with concurrent *TERT* and *BRAF V600E* mutations. However, inconsistent results have been reported by other researchers who could not find a particular relationship or association between the two mutations [14,34,35].

One patient in our sample had simultaneous *PIK3CA/EIF1AX* mutations and nonaggressive disease on final pathology. The role of these specific mutations has yet to be elucidated. The literature proposes that the occurrence of *PIK3CA* mutations may be less useful than *TERT* promoter mutations for assessing the risk of anaplastic transformation in papillary carcinoma [19]. Nonetheless, García-Rostán et al. suggested that *PIK3CA* mutations are more common in anaplastic thyroid cancer (5–25%) than in less aggressive thyroid carcinomas (0–5% in papillary thyroid cancer) [36].

Molecular testing can effectively impact decision making regarding the extent of thyroidectomy required. It has been shown in the literature that utilizing molecular testing in thyroid cancer can help guide the optimal surgery in 91.86% of patients, compared to 61.11% of patients who did not undergo molecular testing [37]. Complications of thyroid surgery, such as hyperparathyroidism, recurrent laryngeal nerve palsy, and lifelong thyroid hormone replacement therapy, can be prevented by reducing overtreatment by thyroidectomy when considered unnecessary [19]. Our findings support the need for more extensive thyroidectomy in nodules with *TERT* promoter mutations and other concurrent mutations, given the high possibility of aggressive disease, but not necessarily in patients with *TERT* promoter mutations alone, given the significantly lower likelihood of aggressive disease. Based on our results, the finding of aggressive disease on postoperative histopathology (and hence the occurrence of a histopathological diagnosis that would warrant at least a total thyroidectomy as a standard treatment approach) was higher in patients with co-existing mutations, as opposed to those with *TERT* promoter mutations alone. Moreover, in cases of single-gene *TERT* promoter mutations, when there are no worrisome clinicodemographic features (e.g., young age or family history of thyroid cancer), one could consider limited treatment with hemithyroidectomy and close observation after shared decision making (i.e., a thorough discussion of treatment options and their benefits and harms with a patient based on best available evidence and a consideration of the patient’s preferences).

Our study has several limitations. First, analysis of other co-mutations and sub-analysis of specific co-mutations was not possible in our study because of the rarity of *TERT* promoter mutations, with its prevalence reported to be between 4.2% and 25% in well-differentiated thyroid cancer. Rent et al. reported the overall prevalence of *TERT* promoter mutations to be 3.5% in their papillary thyroid carcinoma patients [35]. Another limitation is that a significant proportion (42%) of patients in our cohort had indeterminate cytology (Bethesda III and IV), where the prevalence of high-risk mutations, such as *TERT* or *BRAF V600E*, is expected to be lower than in patients with suspicious or malignant cytology (Bethesda IV and VI), where molecular testing is less commonly performed. This is also reflected in our results, which show that 22.2% of Bethesda III/IV cases were aggressive cancers, while 77.8% of Bethesda V/VI cases were aggressive cancers (Table 2, *p* = 0.001). Finally, the detection of GC-rich *TERT* promoter regions by molecular profiling is difficult in general, which may further contribute to the low detection rate.

Other limitations of our study were the small sample size and the high degree of selection bias based on including a nonrandomized sample. Moreover, most patients undergoing thyroid surgery did not undergo molecular testing. Additionally, the retrospective nature of our study may preclude the generalization of our significant results; however, we consider that our results should be perceived as potential findings based on which larger-scale prospective studies can be conducted. Future genetic discoveries will likely result in more accurate molecular panels, and the ongoing understanding of carcinogenesis will allow clinicians to develop more robust management plans and disease predictions.

## 5. Conclusions

In conclusion, thyroid nodules positive for *TERT* promoter mutations concurrent with other mutations had more aggressive malignancies when compared to those positive for *TERT* promoter mutations alone. Moreover, not all patients with a *TERT* promoter mutation alone had a malignancy. Our findings challenge the generalized belief that *TERT* promoter mutations are an independent indicator of aggressive disease. The finding that the aggressiveness of a thyroid tumor with a *TERT* promoter mutation may be dependent on the presence of concurrent mutations is an important concept for thyroid specialists to consider when counseling patients regarding risk stratification, management, and extent of surgery.

## Figures and Tables

**Table 1:** Baseline characteristics.

Factor	Number of Patients (*n* = 24)
Age (years), mean (SD)	68 (10)
Female sex, *n* (%)	18 (75%)
Dominant nodule size (cm), mean (SD)	3.7 (1.7)
Bethesda category, *n* (%)	
3	3 (12.5%)
4	7 (29.2%)
5	1 (4.2%)
6	13 (54.2%)
Molecular profile testing, *n* (%)	
Mutations in *TERT* promoter alone	7 (29.2%)
Mutations in *TERT* promoter with other mutations	17 (70.8%)
Thyroidectomy, *n* (%)	
Hemithyroidectomy	3 (12.5%)
Total thyroidectomy	20 (83.3%)
Completion thyroidectomy	1 (4.2%)
Histopathology, *n* (%)	
Benign	1 (4.2%)
Malignant	23 (95.8%)
Papillary carcinoma	16 (66.7%)
Follicular carcinoma	0 (0%)
Hürthle cell carcinoma	3 (12.5%)
Poorly differentiated carcinoma	4 (16.7%)
Aggressive thyroid carcinoma **	18 (78.3%)
Central neck lymph node metastasis	11 (47.8%)
Distant metastasis	2 (8.7%)

SD: standard deviation; ** Aggressive thyroid cancer included patients with aggressive variants of papillary carcinoma (tall cell, hobnail, solid, diffuse sclerosing), poorly differentiated carcinoma, extensive vascular invasion, gross extrathyroidal extension, and regional or distant metastases.

**Table 2:** Association between disease aggressiveness, sex, Bethesda category, and molecular profile.

	Aggressive (*n* = 18)	Not Aggressive (*n* = 6)	Fisher’s Exact Test (Two-Tailed)
Sex, *n* (%)			*p* = 0.277
Female	12 (66.7%)	6 (100%)	
Male	6 (33.3%)	0 (0%)	
Bethesda category, *n* (%)			*p* = 0.001
Bethesda 3 Bethesda 4	1 (5.6%) 3 (16.7%)	2 (33.3%) 4 (66.7%)	
Bethesda 5 or 6	14 (77.8%)	0 (0%)	
Molecular profile, *n* (%)			*p* = 0.038
*TERT* promoter with another mutation	15 (83.3%)	2 (33.3%)	
* BRAF V600E*	12 (80%)	0 (0%)	
* HRAS/NRAS*	3 (20%)	1 (50%)	
* PIK3CA/EIF1AX*	0 (0%)	1 (50%)	
*TERT* promoter alone	3 (16.7%)	4 (66.7%)	

## Data Availability

The data sets used and/or analyzed during this study are available from the corresponding author upon reasonable request.

## References

[1] Rahib L., Smith B.D., Aizenberg R., Rosenzweig A.B., Fleshman J.M., Matrisian L.M. (2014). Projecting cancer incidence and deaths to 2030: The unexpected burden of thyroid, liver, and pancreas cancers in the United States. Cancer Res..

[2] Bates M.F., Lamas M.R., Randle R.W., Long K.L., Pitt S.C., Schneider D.F., Sippel R.S. (2018). Back so soon? Is early recurrence of papillary thyroid cancer really just persistent disease?. Surgery.

[3] American Thyroid Association. https://www.thyroid.org/patient-thyroid-information/ct-for-patients/volume-8-issue-4/vol-8-issue-4-p-11/.

[4] National Cancer Institute. https://seer.cancer.gov/statfacts/html/thyro.html.

[5] Chen T., Gilfix B.M., Rivera J., Sadeghi N., Richardson K., Hier M.P., Forest V.I., Fishman D., Caglar D., Pusztaszeri M. (2020). The Role of the ThyroSeq v3 Molecular Test in the Surgical Management of Thyroid Nodules in the Canadian Public Health Care Setting. Thyroid.

[6] Lupo M.A., Walts A.E., Sistrunk J.W., Giordano T.J., Sadow P.M., Massoll N., Campbell R., Jackson S.A., Toney N., Narick C.M. (2020). Multiplatform molecular test performance in indeterminate thyroid nodules. Diagn. Cytopathol..

[7] Ozgursoy O.B., Eisele D.W., Tufano R.P. (2014). The prognostic implications from molecular testing of thyroid cancer. Otolaryngol. Clin. N. Am..

[8] Zhao L., Wang L., Jia X., Hu X., Pang P., Zhao S., Wang Y., Wang J., Zhang Y., Lyu Z. (2020). The Coexistence of Genetic Mutations in Thyroid Carcinoma Predicts Histopathological Factors Associated With a Poor Prognosis: A Systematic Review and Network Meta-Analysis. Front. Oncol..

[9] Liu R., Xing M. (2016). TERT promoter mutations in thyroid cancer. Endocr. Relat. Cancer.

[10] Lorbeer F.K., Hockemeyer D. (2020). TERT promoter mutations and telomeres during tumorigenesis. Curr. Opin. Genet. Dev..

[11] Heidenreich B., Rachakonda P.S., Hemminki K., Kumar R. (2014). TERT promoter mutations in cancer development. Curr. Opin. Genet. Dev..

[12] Bell R.J., Rube H.T., Xavier-Magalhães A., Costa B.M., Mancini A., Song J.S., Costello J.F. (2016). Understanding TERT Promoter Mutations: A Common Path to Immortality. Mol. Cancer Res..

[13] Liu X., Bishop J., Shan Y., Pai S., Liu D., Murugan A.K., Sun H., El-Naggar A.K., Xing M. (2013). Highly prevalent TERT promoter mutations in aggressive thyroid cancers. Endocr. Relat. Cancer.

[14] Chen B., Shi Y., Xu Y., Zhang J. (2021). The predictive value of coexisting BRAFV600E and TERT promoter mutations on poor outcomes and high tumour aggressiveness in papillary thyroid carcinoma: A systematic review and meta-analysis. Clin. Endocrinol..

[15] Bournaud C., Descotes F., Decaussin-Petrucci M., Berthiller J., de la Fouchardière C., Giraudet A.L., Bertholon-Gregoire M., Robinson P., Lifante J.C., Lopez J. (2019). TERT promoter mutations identify a high-risk group in metastasis-free advanced thyroid carcinoma. Eur. J. Cancer.

[16] Haugen B.R., Alexander E.K., Bible K.C., Doherty G.M., Mandel S.J., Nikiforov Y.E., Pacini F., Randolph G.W., Sawka A.M., Schlumberger M. (2016). 2015 American Thyroid Association Management Guidelines for Adult Patients with Thyroid Nodules and Differentiated Thyroid Cancer: The American Thyroid Association Guidelines Task Force on Thyroid Nodules and Differentiated Thyroid Cancer. Thyroid.

[17] Bullock M., Ren Y., O’Neill C., Gill A., Aniss A., Sywak M., Sidhu S., Delbridge L., Learoyd D., de Vathaire F. (2016). TERT promoter mutations are a major indicator of recurrence and death due to papillary thyroid carcinomas. Clin. Endocrinol..

[18] Yin D.T., Yu K., Lu R.Q., Li X., Xu J., Lei M., Li H., Wang Y., Liu Z. (2016). Clinicopathological significance of TERT promoter mutation in papillary thyroid carcinomas: A systematic review and meta-analysis. Clin. Endocrinol..

[19] Ebina A., Togashi Y., Baba S., Sato Y., Sakata S., Ishikawa M., Mitani H., Takeuchi K., Sugitani I. (2020). TERT Promoter Mutation and Extent of Thyroidectomy in Patients with 1-4 cm Intrathyroidal Papillary Carcinoma. Cancers.

[20] Oishi N., Kondo T., Ebina A., Sato Y., Akaishi J., Hino R., Yamamoto N., Mochizuki K., Nakazawa T., Yokomichi H. (2017). Molecular alterations of coexisting thyroid papillary carcinoma and anaplastic carcinoma: Identification of TERT mutation as an independent risk factor for transformation. Mod. Pathol..

[21] Vuong H.G., Altibi A.M.A., Duong U.N.P., Hassell L. (2017). Prognostic implication of BRAF and TERT promoter mutation combination in papillary thyroid carcinoma-A meta-analysis. Clin. Endocrinol..

[22] Mascarella M.A., Peeva M., Forest V.I., Pusztaszeri M.P., Avior G., Tamilia M., Mlynarek A.M., Hier M.P., Payne R.J. (2022). Association of Bethesda category and molecular mutation in patients undergoing thyroidectomy. Clin. Otolaryngol..

[23] Vinagre J., Almeida A., Pópulo H., Batista R., Lyra J., Pinto V., Coelho R., Celestino R., Prazeres H., Lima L. (2013). Frequency of TERT promoter mutations in human cancers. Nat. Commun..

[24] Liu X., Qu S., Liu R., Sheng C., Shi X., Zhu G., Murugan A.K., Guan H., Yu H., Wang Y. (2014). TERT promoter mutations and their association with BRAF V600E mutation and aggressive clinicopathological characteristics of thyroid cancer. J. Clin. Endocrinol. Metab..

[25] Wang N., Liu T., Sofiadis A., Juhlin C.C., Zedenius J., Höög A., Larsson C., Xu D. (2014). TERT promoter mutation as an early genetic event activating telomerase in follicular thyroid adenoma (FTA) and atypical FTA. Cancer.

[26] Prete A., Borges de Souza P., Censi S., Muzza M., Nucci N., Sponziello M. (2020). Update on Fundamental Mechanisms of Thyroid Cancer. Front. Endocrinol..

[27] Macerola E., Poma A.M., Vignali P., Basolo A., Ugolini C., Torregrossa L., Santini F., Basolo F. (2021). Molecular Genetics of Follicular-Derived Thyroid Cancer. Cancers.

[28] Silaghi C.A., Lozovanu V., Georgescu C.E., Georgescu R.D., Susman S., Năsui B.A., Dobrean A., Silaghi H. (2021). Thyroseq v3, Afirma GSC, and microRNA panels versus previous molecular tests in the preoperative diagnosis of indeterminate thyroid nodules: A systematic review and meta-analysis. Front. Endocrinol..

[29] Rajab M., Payne R.J., Forest V.I., Pusztaszeri M. (2022). Molecular Testing for Thyroid Nodules: The Experience at McGill University Teaching Hospitals in Canada. Cancers.

[30] Koshkina A., Fazelzad R., Sugitani I., Miyauchi A., Thabane L., Goldstein D.P., Ghai S., Sawka A.M. (2020). Association of Patient Age With Progression of Low-risk Papillary Thyroid Carcinoma Under Active Surveillance: A Systematic Review and Meta-analysis. JAMA Otolaryngol. Head Neck Surg..

[31] Miyauchi A., Kudo T., Ito Y., Oda H., Sasai H., Higashiyama T., Fukushima M., Masuoka H., Kihara M., Miya A. (2018). Estimation of the lifetime probability of disease progression of papillary microcarcinoma of the thyroid during active surveillance. Surgery.

[32] Medici M., Kwong N., Angell T.E., Marqusee E., Kim M.I., Frates M.C., Benson C.B., Cibas E.S., Barletta J.A., Krane J.F. (2015). The Variable Phenotype and Low-Risk Nature of RAS-Positive Thyroid Nodules. BMC Med..

[33] Radkay L.A., Chiosea S.I., Seethala R.R., Hodak S.P., LeBeau S.O., Yip L., McCoy K.L., Carty S.E., Schoedel K.E., Nikiforova M.N. (2014). Thyroid Nodules With KRAS Mutations Are Different From Nodules With NRAS and HRAS Mutations With Regard to Cytopathologic and Histopathologic Outcome Characteristics. Cancer Cytopathol..

[34] Jin L., Chen E., Dong S., Cai Y., Zhang X., Zhou Y., Zeng R., Yang F., Pan C., Liu Y. (2016). BRAF and Tert Promoter Mutations in the Aggressiveness of Papillary Thyroid Carcinoma: A Study of 653 Patients. Oncotarget.

[35] Ren H., Shen Y., Hu D., He W., Zhou J., Cao Y., Mao Y., Dou Y., Xiong W., Xiao Q. (2018). Co-Existence of BRAFV600E and TERT Promoter Mutations in Papillary Thyroid Carcinoma Is Associated with Tumor Aggressiveness, but Not with Lymph Node Metastasis. Cancer Manag. Res..

[36] García-Rostán G., Costa A.M., Pereira-Castro I., Salvatore G., Hernandez R., Hermsem M.J., Herrero A., Fusco A., Cameselle-Teijeiro J., Santoro M. (2005). Mutation of the PIK3CA Gene in Anaplastic Thyroid Cancer. Cancer Res..

[37] Hier J., Avior G., Pusztaszeri M., Krasner J.R., Alyouha N., Forest V.I., Hier M.P., Mlynarek A., Richardson K., Sadeghi N. (2021). Molecular Testing for Cytologically Suspicious and Malignant (Bethesda V and VI) Thyroid Nodules to Optimize the Extent of Surgical Intervention: A Retrospective Chart Review. J. Otolaryngol. Head Neck Surg..

